# The Forgotten Joint Score-12 in Swedish patients undergoing knee arthroplasty: a validation study with the Knee Injury and Osteoarthritis Outcome Score (KOOS) as comparator

**DOI:** 10.1080/17453674.2019.1689327

**Published:** 2019-11-12

**Authors:** Siri Heijbel, Josefine E Naili, Axel Hedin, Annette W-Dahl, Kjell G Nilsson, Margareta Hedström

**Affiliations:** a Department of Clinical Science Intervention and Technology, Karolinska Institutet, Stockholm;; b Department of Women’s and Children’s Health, Karolinska Institutet, Stockholm;; c Lund University, Department of Clinical Sciences Lund, Skåne University Hospital, Department of Orthopedics, Lund;; d The Swedish Knee Arthroplasty Register;; e Department of Surgical and Perioperative Sciences, Orthopedics, Umeå University, Umeå;; f Reconstructive Orthopedics, Karolinska University Hospital Huddinge, Stockholm, Sweden

## Abstract

Background and purpose — Having patients self-evaluate the outcome is an important part of the follow-up after knee arthroplasty. The Forgotten Joint Score-12 (FJS-12) introduced joint awareness as a new approach, suggested to be sensitive enough to differentiate well-functioning patients. This study evaluated the Swedish translation of the FJS-12 and investigated the validity, reliability, and interpretability in patients undergoing knee arthroplasty

Patients and methods — We included 109 consecutive patients 1 year after primary knee arthroplasty to assess construct validity (Pearson’s correlation coefficient, r), internal consistency (Cronbach’s alpha [CA]), floor and ceiling effects, and score distribution. The Knee injury and Osteoarthritis Outcome Score (KOOS) was the comparator instrument for the analyses. Further, 31 patients preoperatively and 22 patients postoperatively were included to assess test–retest reliability (intraclass correlation coefficient [ICC]).

Results — Construct validity was moderate to excellent (r = 0.62–0.84). The FJS-12 showed a high degree of internal consistency (CA = 0.96). The ICC was good preoperatively (0.76) and postoperatively (0.87). Ceiling effects were 2.8% in the FJS-12 and ranging between 0.9% and 10% in the KOOS.

Interpretation — The Swedish translation of the FJS-12 showed good validity and reliability and can be used to assess outcome after knee arthroplasty. Moreover, the FJS-12 shows promising results in its ability to differentiate well-functioning patients. Future studies on unidimensionality, scale validity, interpretability, and responsiveness are needed for a more explicit analysis of the psychometric properties.

Traditionally, the evaluation of outcome following knee arthroplasty has focused to involve objective parameters, such as the range of motion or the risk for revision surgery over a 10-year period. In line with patient-centered health care, supplementary evaluation with subjective parameters using patient-reported outcome measures (PROMs) has increased in popularity during the last decades (Rolfson et al. 2016). The Swedish Knee Arthroplasty Register (SKAR) collects a set of questionnaires that, among other measurements, includes the disease specific PROM Knee injury and Osteoarthritis Outcome Score (KOOS). The KOOS is a well-established 42-item PROM, which address issues of pain and other symptoms along with functionality in daily living, sports and recreational activities, and perceived knee-related quality of life (Roos et al. 1998, Roos and Toksvig-Larsen 2003).

Joint awareness was introduced by Behrend et al. (2012) as a new approach for assessment of outcome following joint arthroplasty. Complete unawareness, put on a par with a joint without problems, has been suggested as the ultimate goal after arthroplasty and, moreover, a sensitive enough measurement to differentiate well-functioning patients (Behrend et al. 2012). A discerning PROM could benefit the evaluation of potential improvements in the surgical techniques or advantageous course of treatments. Such characteristics might become more relevant in the future, as the indications for knee arthroplasty extend and the number of younger patients increases along with the total number of annually performed surgeries (Nemes et al. 2015).

The Forgotten Joint Score-12 (FJS-12) is a concise and user-friendly 12-item PROM that evaluates patients’ ability to forget their joint in daily life. The FJS-12 has previously been translated into several languages with promising results regarding the validity and reliability after knee arthroplasty (Behrend et al. 2012, Thompson et al. 2015, Baumann et al. 2016, Shadid et al. 2016, Thomsen et al. 2016, Cao et al. 2017, Hamilton et al. 2017, Robinson et al. 2018). However, the FJS-12 has not yet been validated for Swedish knee arthroplasty patients.

We evaluated the Swedish translation of the FJS-12 and assessed the validity, reliability, and interpretability in patients undergoing knee arthroplasty, more specifically by assessing the construct validity, internal consistency, test–retest reliability, and floor and ceiling effects. Furthermore, we compared floor and ceiling effects and score distribution between the FJS-12 and the KOOS.

## Patients and methods

### Content validity of the translated version

The developers of the FJS-12 provided a Swedish-language version of the questionnaire, which had been translated in accordance with the report “Principles of Good Practice for the Translation and Cultural Adaption Process for Patient Reported Outcomes (PRO) Measures” (Wild et al. 2005). The Swedish translation was tested in a group of 19 patients with verified knee osteoarthritis at the Departments of Orthopedics at Nyköping and Umeå University Hospitals. The patients were interviewed regarding their perception and the relevance of the FJS-12. Thereafter, all items were tested in their final form and an updated version of the FJS-12 was sent back and accepted by the developers.

### Study setting and patients

This validation study was performed at the Department of Reconstructive Orthopedics at Karolinska University Hospital Huddinge in Stockholm, Sweden. All Swedish-speaking patients, regardless of underlying diagnosis, who underwent primary knee arthroplasty (unilateral knee arthroplasty (UKA) or total knee arthroplasty (TKA)) between June 1, 2016 and December 31, 2017, were eligible for inclusion. Patients were excluded if more than 4 items were missing in the FJS-12, in accordance with Behrend et al. (2012), and if the KOOS data provided by the SKAR were incomplete.

### Data collection

The clinics’ routine 1 year after knee arthroplasty is to provide a set of questionnaires (including the KOOS) to patients by postal mail, which is subsequently reported to the SKAR. To carry through this study, the FJS-12 was added to the regular set of questionnaires in 2017 and collected until March 2019. The FJS-12 data were extracted manually from completed questionnaires returned to the clinic. The KOOS data, sex, age at surgery, BMI, and ASA classification were extracted from the SKAR.

Furthermore, for assessment of test–retest reliability, there were 31 patients preoperatively and 22 patients 1 year postoperatively who received and completed the FJS-12 twice with an interval of at least 2 weeks. No upper time limit for completion was determined.

### FJS-12

The FJS-12 consists of 12 items that are related to joint awareness in daily life (Behrend et al. 2012). Each item is answered within a 5-point Likert scale with the following response options: never (0 p); almost never (1 p); seldom (2 p); sometimes (3 p); and mostly (4 p). The initial raw data were converted to a scale ranging from 0 to 100 (worst to best), by dividing the summarized score by the number of completed items, which subsequently was multiplied by 25 and thereafter subtracted from 100.

## KOOS

The KOOS consists of 42 items divided into 5 domains: Symptoms, Pain, Function in Daily Life (ADL), Function in Sport and Recreational Activities (Sport/Rec), and Knee-related Quality of Life (QoL) (Roos et al. 1998). Each item is answered within a 5-point Likert scale. Initial raw data from each of the domains had been converted to a scale ranging from 0 to 100 (worst to best).

### Statistics

Construct validity was assessed by Pearson’s correlation coefficient (r). The FJS-12 was correlated to each of the KOOS domains. The correlations were classified as little or no correlation (0–0.25); fair degree of correlation (0.25–0.50); moderate to good correlation (0.50–0.75); and very good to excellent correlation (0.75–1) (Dawson and Trapp 2004). The correlations were expected to be at least 0.5 to all of the KOOS domains.

Internal consistency was assessed by Cronbach’s alpha, which was considered as adequate if the value was between 0.70 and 0.95 (Terwee et al. 2007). Test–retest reliability was assessed by calculating the intra-class correlation coefficient (ICC) for a 2-way random effect model with measures of absolute agreement. The ICC was calculated for each group (preoperatively and postoperatively) and was thereafter classified as poor (0–0.5), moderate (0.5–0.75), good (0.75–0.9), or excellent (0.9–1) (Koo and Li 2016).

Floor and ceiling effects were assessed and compared for the FJS-12 and for each of the KOOS domains. Floor and ceiling effects were determined to be pronounced if the percentage of patients, with a total score of either 0 or 100, was > 15% respectively (Terwee et al. 2007). Score distributions were investigated with histograms.

For test of normality, the Shapiro–Wilk test was used. Mean values and standard deviation (SD) are given for normally distributed data and median and range are given, in addition, for non-normally distributed data. Construct validity and test–retest reliability are presented with 95% confidence interval (CI). The statistical analyses were performed with SPSS Statistics 25.0 (IBM Corp, Armonk, NY, USA) and Microsoft Excel (Microsoft Corp, Redmond, WA, USA).

### Ethics, funding and potential conflicts of interests

The study was approved by the Regional Ethical Review Board in Stockholm (2014/1895-31/3) and was supported by grants provided by Region Stockholm (NSV project). No competing interests were declared.

## Results

### Content validity

After patient debriefing in the test group, item number 12 “… when you are doing your favorite sport?” was changed to “… when you exercise?”. All other questions were regarded as relevant and easily understood. In the phrase “Are you aware of your affected joint…”, the word “affected” was originally translated to the Swedish word “drabbad”. The word “drabbad” is something undesirable or unpleasant and was therefore changed to the more neutral word “berörd”.

### Validation process

During the study period there were 177 primary knee arthroplasties performed, from which 145 patients returned the questionnaires to the clinic. There were 36 patients with incomplete questionnaires excluded from the study, leaving a set of 109 (62%) questionnaires available for the analyses. From the included questionnaires 11 items were missing, distributed over 6 of the questions. The mean time from surgery to completion of the questionnaires was 13.5 months (10–20). From the included patients there were 86 who underwent TKA and 23 who underwent UKA. Demographic data and scores of the FJS-12 and the KOOS are summarized in Table 1.

**Table 1.** Demographic data, postoperative mean (SD) and median (range) score of the FJS-12 and the KOOS domains

Characteristics	TKA	UKA	All patients
Number of patients (%)	86 (79)	23 (21)	109 (100)
Women, n (%)	51	14	65 (60)
Age at surgery			
Mean (SD)	69 (9)	71 (7)	69 (9)
Mean BMI (SD)	30 (5)	28 (3)	30 (7)
ASA classification, n (%)			
1	6	3	9 (8)
2	42	11	53 (49)
3	37	9	46 (42)
N/A	1	–	1 (1)
Median score (range)			
FJS-12	33 (0–100)	40 (0–100)	35 (0–100)
KOOS Symptoms	75 (25–100)	75 (46–100)	75 (25–100)
KOOS Pain	83 (5–100)	81 (31–100)	83 (6–100)
KOOS ADL	79 (10–100)	78 (36–100)	78 (10–100)
KOOS Sport/Rec	25 (0–100)	40 (0–95)	30 (0–100)
KOOS QoL	53 (0–100)	63 (19–100)	56 (0–100)
Mean score (SD)			
FJS-12	37 (30)	50 (33)	40 (31)
KOOS Symptoms	72 (18)	79 (15)	74 (18)
KOOS Pain	76 (23)	79 (20)	77 (22)
KOOS ADL	70 (25)	77 (21)	72 (24)
KOOS Sport/Rec	32 (29)	43 (28)	34 (29)
KOOS QoL	57 (26)	64 (25)	58 (26)

BMI = Body Mass Index;

ASA = American Society of Anesthesiologists;

FJS-12 = Forgotten Joint Score-12;

KOOS = Knee injury and Osteoarthritis Outcome Score;

ADL = Functions in daily life;

Sport/Rec = Functions in sport and recreational activities;

QoL = Knee-related quality of life.

### Construct validity

Pearson’s correlation coefficient of the FJS-12 to the KOOS ranged from moderate to excellent: Symptoms, r = 0.62 (CI 0.48–0.71); Pain, r = 0.72 (CI 0.63–0.79); ADL, r = 0.74 (CI 0.66–0.80); Sport/Rec, r = 0.65 (CI 0.55–0.74) and QoL, r = 0.84 (CI 0.79–0.88).

### Reliability and reproducibility

The interrelatedness amongst the items of FJS-12 was investigated and a high level of internal consistency was found (Cronbach’s alpha 0.96). The test–retest reliability of the FJS-12 was classified as good with an ICC of 0.76 (CI 0.55–0.87) in the preoperative group and 0.87 (CI 0.72–0.94) in the postoperative group.

### Interpretability

**Figure 1.**
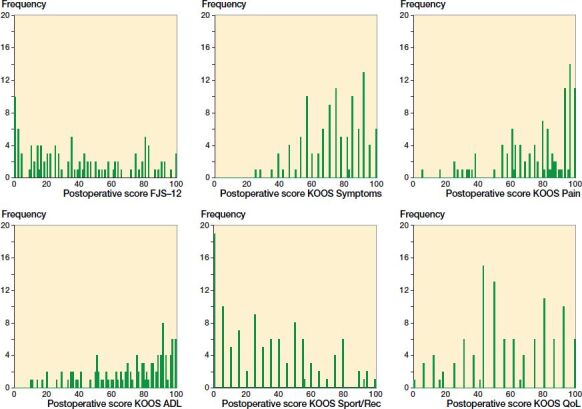
Distribution of scores 1 year after knee arthroplasty in FJS-12 and KOOS domains: Symptoms; Pain; Functions in daily life (ADL); Functions in sport and recreational activities (Sport/Rec), and Knee-related quality of life (QoL).

There were no pronounced ceiling effects in the FJS-12 or in the KOOS whereas a pronounced floor effect (17%) was found in the domain Sport/Rec (Table 2). Figure 1 presents the score distribution over the scales in the FJS-12 and the KOOS domains.

**Table 2.** Number (%) of patients with floor effect (0 points), ceiling effect (100 points) out of 109 patients

Questionnaire	Floor effect	Ceiling effect
FJS-12	10 (9)	3 (3)
KOOS Symptoms	0	6 (6)
KOOS Pain	0	11 (10)
KOOS ADL	0	6 (6)
KOOS Sport/Rec	19 (17)	1 (1)
KOOS QoL	1 (1)	6 (6)

For abbreviations, see Table 1.

## Discussion

In the FJS-12 the patients are asked to rate their level of joint awareness during common activities in daily life. The findings in this study suggest that the FJS-12 has good validity and reliability. Furthermore, when comparing score distribution and floor and ceiling effects between the FJS-12 and the KOOS, the results indicates that the FJS-12 is an appropriate PROM to use for differentiation of well-functioning patients. To further investigate and strengthen the validity of the FJS-12, future studies exploring the unidimensionality, scale validity, interpretability, and responsiveness are needed.

### Construct validity

Joint awareness has been suggested to integrate a variety of variables such as pain, stiffness, and functionality in daily living (Behrend et al. 2012). Therefore, the correlations between the FJS-12 and the KOOS are expected to be at least moderate but not absolute. We found moderate to excellent correlations (r = 0.62–0.84), findings that are similar to those found by Cao et al. (2017) and Thompson et al. (2015), who reported strong correlations between the FJS-12 and the KOOS. We found the strongest correlation between the FJS-12 and the KOOS domain QoL. This particular domain includes items of a more general character, compared with the more explicitly characterized items in the other domains, and may in that aspect be more like the FJS-12.

The conceptual generality of joint awareness may be a disadvantage with the FJS-12. Although the versatility is likely to provide a sensitive measurement of both improvement and deterioration, as well as differences that can occur between patients, it may also allow for psychological factors to influence the scores, such as insecurity and fear of complications (Loth et al. 2018).

### Reliability and reproducibility

If the value of Cronbach’s alpha exceeds the upper limit for positive rating (0.95) according to Terwee et al. (2007), this could indicate a redundancy between the items. In this study, the value of Cronbach’s alpha was 0.96, similar to the majority of the previous FJS-12 validation studies (Behrend et al. 2012, Baumann et al. 2016, Thomsen et al. 2016, Hamilton et al. 2017, Robinson et al. 2018). The high value of Cronbach’s alpha may be due to the similarity of the questions, since they all begin with the same phrase, “Are you aware of your joint…”. It may be that some patients conceptualize joint awareness as something constant rather than situation dependent.

The ICC was investigated for both the preoperative patients and the postoperative patients separately, since the value of the coefficient depend partly on the sample variation (Terwee et al. 2007). The results were classified as good test-retest reliability, which is similar to previous findings (Thompson et al. 2015, Thomsen et al. 2016, Cao et al. 2017, Robinson et al. 2018). Furthermore, a true value of the ICC requires that the patients are free from clinical changes that interfere with the measurements. Given this, the ICC that was calculated in the postoperative patient group (0.87) may be closer to the true value compared with the ICC in the preoperative patient group (0.76).

### Interpretability

A pronounced floor effect was found in the KOOS domain Sport/Rec (see Table 2). This may be caused by specific movements, i.e., squatting, running, kneeling, jumping, and pivoting/twisting, that are difficult to perform by a large number of patients who have undergone knee arthroplasty. Previous reports of the FJS-12 ceiling effects after knee arthroplasty range from 0% to 9.2% (Baumann et al. 2016, Thienpont et al. 2016, Hamilton et al. 2017, Robinson et al. 2018). The present study showed that the FJS-12 had lower ceiling effects than the KOOS (Table 2), which has similarly been found in other studies (Thompson et al. 2015, Thienpont et al. 2016). Our findings on ceiling effects (2.8%) are considerably lower compared with recently published results (21%) by Larsson et al. (2019) for the FJS-12 in patients undergoing total hip arthroplasty (THA). This might limit the usability of the FJS-12 in THA patients, which has previously been discussed by Thienpont et al. (2016), who found a higher ceiling effect in patients undergoing THA compared with TKA.

Figure 1 suggests that the scores have a greater spread over the scale in the FJS-12 compared with the KOOS. This may be because complete unawareness of a knee with a joint implant is more difficult to achieve, compared with an outcome such as a satisfying degree of pain relief. Our findings indicate a comprehensiveness in the FJS-12 that enables detection of differences between patients, which may indicate that the FJS-12 provide attributes with high discriminatory power in this patient group.

### Feasibility

The conciseness of the FJS-12 may be more appealing for patients than the extensiveness of the KOOS. Cao et al. (2017) reported a mean completion time of 85 seconds, which is considerably shorter than the approximate 10 minutes’ completion time for the KOOS. Although joint awareness may be conceptually challenging for some patients to interpret, the opinions that were expressed in the pilot study regarding the relevance and understanding of the FJS-12 were positive.

### Strengths and limitations

A strength of the present study is that the sample size for construct validity, internal consistency, and floor and ceiling effects met the criteria proposed by Terwee et al. (2007). Further, the study comprises a consecutive group of patients and all arthroplasties were performed according to the clinics’ everyday routine, altogether minimizing the risk of selection bias in the sample. However, the sample could have been affected by the response rate.

Further, this was a university hospital-based study while a large proportion of the knee arthroplasties in the Stockholm region are provided by private-driven healthcare facilities, which may affect the generalizability of the score distribution and floor and ceiling effects. While the mean age in our study sample was comparable to the mean age of the Swedish knee arthroplasty patients, the mean KOOS scores were somewhat lower (SKAR 2018). This may be explained by the higher proportion of patients with ASA ≥ 3 and BMI 35+ who were referred to the hospital concerned, as compared with the general knee arthroplasty population (SKAR 2018).

We did not investigate the structural validity of the FJS-12, which decreases the reliability of the internal consistency measurement (Terwee et al. 2007). Although Cronbach’s alpha indicates that the items are related to one another, the internal consistency of the FJS-12 is not completely explored unless the items have been evaluated with factor analysis.

To avoid the ICC being affected by the heterogeneity of the preoperative and postoperative patient groups, we performed separate calculations for each, which resulted in rather small sample sizes. When the test–retest reliability is evaluated, there is no consensus regarding the exact time period between the first and second response. In this study, a period of at least 2 weeks was chosen to prevent recall bias. However, there was no upper time limit for completion, which may have falsely lowered the result for the ICC analysis due to possible clinical changes in the patients.

## Conclusion

Our study shows that the FJS-12 has good validity and reliability and may be used after knee arthroplasty in clinical practice, or as a supplement to the KOOS in the SKAR. Furthermore, the results indicate that the FJS-12 may provide attributes that differentiate well-functioning patients. This might be especially useful for future clinical research on improving the treatment for patients who undergo knee arthroplasty.

SH drafted the manuscript, performed the statistical analyses, and managed the data. JEN drafted the manuscript. AH managed the data and was involved in conception of the pilot study. AWD drafted the manuscript. KGN was involved in conception of the pilot study. MH was involved in in conception and design, drafted the manuscript, and conception of the pilot study. All authors have made substantial contributions in the interpretation of data and revision of the manuscript.
